# Inflammation-Related Effects of Diesel Engine Exhaust Particles: Studies on Lung Cells *In Vitro*

**DOI:** 10.1155/2013/685142

**Published:** 2013-02-14

**Authors:** P. E. Schwarze, A. I. Totlandsdal, M. Låg, M. Refsnes, J. A. Holme, J. Øvrevik

**Affiliations:** Division of Environmental Medicine, Norwegian Institute of Public Health, Lovisenberggaten 8, 0403 Oslo, Norway

## Abstract

Diesel exhaust and its particles (DEP) have been under scrutiny for health effects in humans. In the development of these effects inflammation is regarded as a key process. Overall, *in vitro* studies report similar DEP-induced changes in markers of inflammation, including cytokines and chemokines, as studies *in vivo*. *In vitro* studies suggest that soluble extracts of DEP have the greatest impact on the expression and release of proinflammatory markers. Main DEP mediators of effects have still not been identified and are difficult to find, as fuel and engine technology developments lead to continuously altered characteristics of emissions. Involved mechanisms remain somewhat unclear. DEP extracts appear to comprise components that are able to activate various membrane and cytosolic receptors. Through interactions with receptors, ion channels, and phosphorylation enzymes, molecules in the particle extract will trigger various cell signaling pathways that may lead to the release of inflammatory markers directly or indirectly by causing cell death. *In vitro* studies represent a fast and convenient system which may have implications for technology development. Furthermore, knowledge regarding how particles elicit their effects may contribute to understanding of DEP-induced health effects *in vivo*, with possible implications for identifying susceptible groups of people and effect biomarkers.

## 1. Introduction

Living close to heavily trafficked roads has been associated with adverse effects on people's health, including increased mortality and morbidity from cardiopulmonary causes [1]. Though the proximity to dense traffic includes exposure to noise and gasses, which may adversely affect health [1, 2], emissions of particle matter (PM) from traffic are estimated to have a major adverse impact on health [1, 3]. Notably, exposure to PM has been associated with adverse effects on the pulmonary as well as cardiovascular system [4, 5]. PM from traffic comprises road dust, vehicle particles, and exhaust particles, which have all been linked to adverse health outcomes [1]. However, most publications focus on the exhaust particles. 

Traditionally diesel engines have mostly been used in heavy duty vehicles. However, according to statistics from the European automobile manufacturers' association (ACEA), there has been a large increase in the percentage of newly registered diesel passenger cars in Western Europe, from 13.8% in 1990 to 50.6% in 2010. This is an intended increase as a means to cut CO_2_ emissions from transport. However, diesel-fuelled vehicles with pre-EURO 5 technologies or the equivalent US standard are known for their higher PM emissions compared to gasoline-fuelled vehicles, and will dominate the car fleets for several years, considering the average age of the vehicles in operation. As a result of the significant contribution of PM emissions from diesel vehicles for the total concentration of PM in ambient air, extensive research on effects of diesel engine exhaust (DEE) and diesel exhaust particles (DEP) has been carried out. 

Inflammation is considered a key step in the development of health effects associated with PM exposure [6–9]. Activated immune cells including neutrophils and macrophages will release cytokines, reactive oxygen species (ROS), lipid mediators, and toxic proteases, which may further amplify and contribute to any DEP-induced epithelial damage. A further increased release of pro-inflammatory mediators from the epithelium and/or induced epithelial cell death may augment as well as prolong the inflammatory reactions, and ultimately result in chronic inflammation if the exposure persists. Furthermore, the increased oxidative stress caused by activated immune cells, may also contribute to DEP-induced DNA damage, and mutations [10]. Notably, chronic inflammation is also a central part of cancer development [11]. In addition to promoting the development of lung diseases, airway inflammation may be a cardiovascular risk factor. Evidence suggests that inflammation in the lung may lead to systemic inflammation, which may increase the susceptibility to acute cardiovascular disease [12–14].

Cell culture models *in vitro* have been used to characterize various toxic effects of DEP including DNA damage, effects on cell proliferation, release of cytokines and chemokines, differentiation and/or capacity of immune cells to defend against infections cytotoxicity (cellular differentiation), and cytotoxicity. Furthermore, such models have been used to investigate effects of various types of DEP and DEP-components, as well as to study more detailed mechanisms of effect. Since DEP may deposit at various places in the airways [15], different lung epithelial cells as well as immune cells and combinations have been used. 

Many factors influence the chemical and physical properties of collected DEP; among them are engine technology, fuel, load, temperature and filtration devices. Thus, a range of different DEP samples with varying composition have been studied. Some studies have also investigated the effect of engine and fuel interventions (Table 1). This paper summarises *in vitro* studies of pro- and anti-inflammatory- and allergy- linked responses of lung cells after exposure to different DEP and associated compounds. Focus will be on mechanisms involved in pro-inflammatory effects. This knowledge will contribute to the understanding of DEP-induced health effects *in vivo* and represent a fast and convenient system for technology development.

## 2. Expression and Release of Cytokines and Chemokines

Inflammatory reactions *in vivo* involve the production and release of a range of signalling molecules such as cytokines, chemokines, and leukotrienes/prostaglandins and adhesion molecules. These molecules operate in a complex network between epithelial cells and immune cells including macrophages, neutrophils, eosinophils, dendritic cells, and Th-cells [6, 44]. Depending on the cytokines and/or chemokines released, different classes of immune cells will be recruited and affect the outcome of the inflammatory response. In mild to moderate allergic asthma related responses eosinophils are the predominant inflammatory cells, whereas episodes of acute exacerbation of asthma tend to be driven by neutrophilic inflammation [45]. Neutrophils are also believed to be the more important for the development of chronic obstructive pulmonary disease (COPD), because of their ability to release elastase which mediates tissue destruction [46]. Eosinophils express CCR3-chemokine receptors and are primarily activated and recruited by chemokines CCL-chemokines such as CCL5/-7/-11/-16/-24 and -26, whereas neutrophils express CXCR1 and -2 receptors and are primarily activated by CXCL-chemokines including CXCL1-3 and -5-8 [47]. Similarly, other classes of immune cells such as dendritic cells, basophils, B-cells, and various T cells express relatively specific sets of chemokine receptors allowing for targeted activation of different types of immune responses.

At a cellular level, the onset of pro-inflammatory reactions tends to start by release of early-responding cytokines such as interleukin (IL)-1*α* and-*β* and tumor necrosis factor (TNF)-*α*. IL-1*α*/-*β* and TNF-*α* tend to be expressed as inactive proforms in resting cells and may be rapidly cleaved and released without requiring activation of the transcriptional machinery. This enables an immediate response upon encounters with inhaled pathogens or xenobiotics. IL-1*α*/-*β* and TNF-*α* subsequently regulate the expression of a variety of secondary cytokines and chemokines, including IL-6 and CXCL8 (IL-8). However, secondary cytokines may also be activated more directly by DEP (independently of IL-1*α*/-*β* and TNF-*α*) through activation of pro-inflammatory signalling pathways within the cells. 

The release of IL-1*β* is tightly controlled, which seems to requires a dual pathway activation process involving transcriptional activation of the IL-1*β* gene leading to formation of pro-IL-1*β* and activation of the NALP3 inflammasome system. This leads to cleavage of the pro-form and subsequent release of active IL-1*β* [48]. Increased expression of pro-IL-1*β* is often found after exposure to lipopolysaccharide (LPS), an inflammatory Toll-like receptor (TLR) agonist from bacterial walls. NAPL3 on the other hand responds to so-called danger-associated molecular patterns (DAMPs) released during cellular necrosis, such as uric acid crystals [49]. However, DAMPs and other NALP3 activators do not seem to induce formation of pro-IL-1*β*. Thus priming with LPS is often required for DAMPs to induce cellular IL-1*β* release. Interestingly, in the absence of LPS, an increased expression of IL-1*β* in alveolar epithelial lung cells (A549) was found in an air-liquid exposure system to freshly generated DEP [42]. Also, in THP-1 monocytes NIST-2975 DEP induced an increase in IL-1*β* release, independently of the presence of LPS [20]. In a coculture of BEAS-2B cells and primary human monocytes, NIST 2975 DEP enhanced the cytokine release of IL-8 after pre-treatment with LPS. [26]. However, this response does not seem to occur through a “classic” activation of the NALP3-inflammasome complex. NIST 2975 DEP is also reported to reduce an IL-1*β*-induced IL-8 response in the BEAS-2B and monocytes co-culture [26]. Furthermore, central elements of the NALP3-inflammasome did not seem to be needed in responses to DEP in transgenic mice [50]. 

TNF-*α* is expressed as a membrane bound proform that is released upon cleavage by the metalloprotease TNF-*α* converting enzyme (TACE), also known as ADAM-17 [51]. Thus, DEP may induce pro-inflammatory effects dependent on TACE-mediated TNF-*α* cleavage. DEP have been reported to induce TNF-*α* responses in primary monocyte-derived macrophages, and cytokine responses in endothelial cells exposed to conditioned media from DEP-exposed macrophages was suppressed by TNF-*α*-inhibition [52]. Diesel-enriched PM has also been found to induce TNF-*α* in monocyte-derived dendritic cells [53]. In contrast, studies in human and murine alveolar macrophages have reported that DEP rather suppress TNF-*α* responses [54–56]. To the best of our knowledge, no effects of DEP or DEP-extracts on TNF-*α* response have been reported from pulmonary epithelial cells. However, DEP have been found to induce TNF-*α* responses in middle-ear epithelial cells [57], but not in human keratinocytes [58]. In total, TNF-*α*-release does not seem to be a prerequisite for DEP to induce pro-inflammatory responses *in vitro*. In line with this, *in vivo* studies with TNF-*α* knock-out mice suggest that DEP-induced inflammation is triggered independently of TNF-*α* [59].

Many *in vitro* studies on lung epithelial cells have reported that DEP induce the release of a number of cytokines and chemokines that are involved in both innate and/or adaptive immunity inflammatory responses [60]. Innate immunity cytokines such as IL-6 and IL-8 have often been included in investigations of DEP effects. In studies with BEAS-2B bronchial epithelial lung cells DEP from a pre-year-2000 engine increased the release of chemokines such as IL-8 [33, 38]. In a comparison of different DEP, the older DEPs from Japan [35] and the NIST 2975 were considerably more potent than the more recently produced DEP from EPA (Table 1). However, not all studies report increased IL-8 levels after DEP exposure, despite increased IL-8 RNA levels [23]. In a broader analysis on the mRNA level of EURO-4 DEP-induced cytokines in BEAS-2B cells, Totlandsdal and coworkers observed increased levels of CCL5 (RANTES), CXCL10, and IL-1*β* among others, in addition to high levels of IL-6 and IL-8 [23]. However, only the last two reached statistical significance. Increased RANTES after DEP-exposure (pre-year-2000 engine) has also been reported by Hashimoto and co-workers [40]. 

Thymic stromal lymphopoietin (TSLP) is an an IL-7-like cytokine which has received considerable attention as a possible “master-switch” in allergic disease. TSLP from epithelial barrier surfaces suppress p40 and induce OX40L expression by dendritic cells (DCs), which suppress Th1 and promote Th2 responses, respectively [61]. Exposure to pre-year-2000 DEP has been shown to induce TSLP release from human bronchial epithelial cells (BEAS-2B) subsequently leading to maturation and polarisation of DCs [41]. The DEP-dependent release of TSLP from the epithelial cells also caused the DCs to express OX40L ligand and Jagged-1 [62]. Thus effects of DEP on epithelial lung cells may promote the induction of Th2 responses, often associated with adaptive immunity and allergic asthma. The ability of DEP to stimulate adaptive immune responses has been corroborated by a study in transgenic mice [63]. 

An increase in granulocyte macrophage colony-stimulating factor (GM-CSF), that stimulates monocyte multiplication, has also been observed after DEP exposure [17, 38]. DEP-enriched particulate matter sampled in a tunnel stimulated cytokine release (IL-12, TNF-*α*, IL-6, and interferon (IFN)-*γ*) from primary monocyte-derived dendritic cells [53]. This indicates that other cell types can contribute to a pro-inflammatory pattern of cytokines and chemokines in concert with macrophages and epithelial cells. Moreover, conditioned medium from macrophages exposed to DEP (NIST 2975) stimulated primary human endothelial cells (HUVEC) to release cytokines and chemokines through a TNF-*α* dependent mechanism, indicating that pulmonary inflammatory markers may influence systemic cells [52]. Similar observations have also been reported from studies with carbon black particles [64]. 

## 3. Other Inflammation-Related Proteins

In addition to cytokines and chemokines, a variety of other signalling molecules are involved in the orchestration of inflammatory responses. ICAM-1 plays a critical role in the adhesion of inflammatory cells to reach the place of an inflammatory response. BEAS-2B cells have been shown to increase expression of this molecule on their surfaces upon exposure to pre-year-2000 DEP [39]. 

Epithelial cells can form a tight monolayer that presents a barrier to prevent the external agents from entering the circulation. NIST DEP can increase transepithelial electrical conductance and loosening of the tight junctions, thus increasing the possibility of leakage of proteins and perhaps particles to the vascular system [34]. In a triple co-culture model (bronchial epithelial 16HBe140 cells, monocyte derived macrophages, and dendritic cells) Lehmann et al. observed that NIST 2975 modulated occludin RNA levels which may have implications for tight junction function. However the effect was only observed at the highest concentration [28]. In this connection, it is interesting to note that NIST 2975 DEP have been reported to increase the release of metalloproteinase MMP-1 from human lung epithelial cells (A549 and NCI-H292). MMP-1 is involved in the degradation of collagen and can thus damage the lung epithelial barrier, probably involving an oxidative mechanism that includes NOX4 [29]. These findings suggest that DEP not only can induce pro-inflammatory responses, but also contribute to structural changes with inflammatory implications.

Prostaglandins function as attractants of inflammatory cells. Studies have found that EURO-4 DEP increased the levels of COX-2 in BEAS-2B cells, a key enzyme in prostaglandin production [23]. This response was also observed with three different DEP (Table 1). DEP have also been reported to enhance COX-2- and prostaglandin E2-responses in monocyte-derived macrophages primed with TLR2 and -4 ligands [65].

Heat shock proteins HSP70 and HSP40 and two other proteins involved in the protein unfolding response were induced after exposure of BEAS-2B cells to pre-year-2000 DEP extracts. This response was accompanied by an increase in IL-6 and IL-8 and possibly related to DEP-induced oxidative stress [36].

Many studies have reported that DEP induce the expression of phase I and II xenobiotic metabolising enzymes through activation of the AhR and Nrf2 transcription factors, respectively [18, 23, 66–68]. Thus, AhR- and Nrf2-regulated gene expression may represent indirect biomarkers of exposure to DEP. Furthermore, phase I enzymes are involved in metabolic activation of various xenobiotics to reactive electrophilic metabolites including ROS, possible triggering molecules of inflammatory reactions following DEP-exposure. This may occur not only directly with regard to triggering of cytokines/chemokine release, but also indirectly via increased cellular toxicity and release of inflammatory DAMP molecules. Thus, changes in the balance between activation and detoxification of the metabolism of xenobiotic in the lung cells may have important inflammatory implications. The phase I enzyme CYP 1A1 is reported to be induced at concentrations of about 0.1 *μ*g/mL or 0.004 *μ*g/cm^2^ [23]. However, the increase in CYP1A1 was greatly reduced at higher concentrations, when inflammatory cytokines and chemokines were prominent [23]. The data indicate a reciprocal relationship between the AhR/Arnt-dependent CYP1A1 induction and cytokine production, apparently because AhR has an inhibitory role in the control of inflammation [69–71]. Also the stimulation of the expression of phase II detoxification enzymes by DEP extracts seemed to reduce the cytokine response [72].

## 4. Particle Considerations 

There seems to be obvious differences in the composition of DEP of different age and source. Some older DEP-preparations are apparently less potent than some newer ones (e.g., EURO 4 DEP), although these differences do not seem to correlate with “classic”, carcinogenic polycyclic aromatic hydrocarbons (PAH) content (unpublished results from our lab). DEP typically consists of agglomerates of primary carbon particles 15–30 nm in diameter and nucleation mode particles of condensed hydrocarbons and sulphate [73]. However, differences in engine and emission-cleansing technologies may affect the ratio of these two main particle fractions in diesel exhaust emissions and has also considerable impact on the amount and composition of chemicals adhered to the surface of the emitted DEP [74]. 

### 4.1. Importance of Soluble Organic Fraction of DEP

In general, both the particulate as well as the organic components of DEP are of importance for DEP-induced effects [75–77]. However, some *in vitro* studies comparing effects induced by organic DEP extracts and corresponding residual particles (subjected to extraction) have demonstrated that the organic fractions of DEP may be of particular importance for pro-inflammatory responses [19, 78, 79]. It is yet unclear to what extent these responses to the organic extracts could be attributed to the presence of specific groups of compounds. Recent studies indicate that polar organic extracts of PM were found to induce cytotoxicity and IL-6 responses in BEAS-2B cells, while nonpolar organic PM-extracts had no apparent effect [80]. In accordance with this, observations from our lab indicate that compounds eluting in the polar fraction of methanol-extracts of DEP may be of particular importance. Notably, the effects of the polar fraction could not be attributed to identified PAH and PAH-derivatives in the extract (Totlandsdal et al., unpublished results).

Suppressive effects of DEP and DEP organic extracts on various immune responses have also been reported. More specifically, DEP and DEP organic extracts have been found to reduce alveolar macrophage function as demonstrated by reduced production of cytokines (IL-1, TNF-*α*) and ROS in response to a variety of biological agents (LPS, interferon-*γ* and bacteria) [54–56, 76]. Different fractions of organic DEP-extracts have been tested for the ability to suppress NO production from BCG-stimulated macrophages [22]. The polar fractions seemed more inhibitory than the less polar fractions [22]. Recent observations from our lab also suggest that high-polar organic DEP-extracts supress IL-8 responses in BEAS-2B cells, despite stimulating IL-6 responses (Totlandsdal et al. unpublished results). Similar findings have been reported for polar extracts of ambient PM [80]. Furthermore, it has been shown that the organic content of DEP, which was in the rank order NIST 2975 < compressor diesel (EPA) < automobile diesel, did not correlate with the immune responses [33]. Thus, obviously there are specific compounds of the organic fraction of DEP, differing with regard to DEP-age, -fuel type, and/or engine, that are of particular importance for cellular responses.

With respect to allergy-related responses there are to our knowledge no *in vitro* studies that investigate the difference between DEP and corresponding DEP organic extracts and residual DEP. However, a study in mice has indicated that the organic fraction rather than the washed particles may be responsible for the enhancement of allergy-related responses, although a role for the solid core of the carbonaceous particles could not be excluded [81]. 

### 4.2. Biodiesel

A major reason for the interest in biodiesel fuel has been environmental benefits in terms of decreased global warming impacts and reduced emissions. Increased use of biodiesel in Europe represents an important step for the European Union in order to meet its emission reduction targets and a variety of biofuels are already being introduced into wider use in heavy-duty diesel engines such as those installed in buses and trucks in cities. However, according to a previous review, biodiesel exhaust emission has been extensively characterized under field and laboratory conditions, but there have been limited studies on the effects of biodiesel exhaust in biologic systems [82].

More recently, it has been demonstrated by human inhalation studies that biodiesel (soy bean ethyl esters, SEE: B50 and B100 (50 and 100% biodiesel, resp.)) was equally or more toxic than fossil diesel in promoting cardiovascular alterations as well as pulmonary and systemic inflammation [83]. Coherent with the inflammatory potential of biodiesel demonstrated in this *in vivo* study, recent *in vitro* work from our lab demonstrates a greater toxic and pro-inflammatory capacity in human bronchial epithelial BEAS-2B cells of DEP with biodiesel (rape seed oil methyl esters, RME) than ordinary DEP, especially in the presence of a diesel particle filter (Gerlofs-Nijland et al. unpublished results). Moreover, extracts of biodiesel (blend of SEE and SME) have been reported to induce cytokine responses in BEAS-2B cells at lower concentrations than extracts of petroleum diesel [32]. In contrast, Jalava and colleagues [84] observed that biodiesel DEP were as potent as ordinary DEP, or less potent, depending on the end point (pro-inflammatory response, cell death, DNA damage, or oxidative potential in a mouse macrophage-like cell line (RAW264.7)). In a separate study, only pure fossil diesel and not B20-blends of RME or animal fat methyl esters (AFME) was found to induce ICAM-1 and VCAM-1 expression in primary human umbilical cord cells (HUVECs), and none of the tested DEPs affected IL-8 and CCL2 expression significantly in the human monocytic cell line, THP-1 [85]. Whether these apparent discrepancies in the pro-inflammatory potential of biodiesel are due to cell specific effects or differences in the chemical composition of the DEPs used in the different studies remains to be clarified. Importantly, as pointed out by Brito and colleagues [83], biodiesel may from a commercial point of view be considered as a cleaner, less toxic, and more biodegradable fuel. Thus, the above findings clearly highlight the importance of further studies to elucidate how and to what extent biodiesel fuels affect pro-inflammatory compared to conventional fossil fuel.

## 5. Biological Mechanisms of DEP-Induced Proinflammatory and Cytotoxic Effects

The genotoxicity of DEP is to a large extent considered to be due to chemicals found in the organic DEP-extracts, such as PAH. In particular, nitro-PAH seem to be important for the mutagenicity of DEP [86, 87]. Also the pro-inflammatory DEP-effects seem mainly to be mediated by constituents in organic DEP-extracts [19, 37, 78, 79]. Most interestingly, DEP with different amount of soluble organic materials may induce IL-8 responses through different mechanisms [33]. DEP with high organic content induced IL-8 through activation of AP-1, while DEP with low organic content induced IL-8 through nuclear factor (NF)-*κ*B. The reason for this difference is unclear, but since activation of the AhR may lead to suppression of NF-*κ*B signaling [69, 71], it is conceivable that the effects could be related to a higher content of AhR-activating compounds in the organic-rich DEP. In any case, the observation is of particular importance as it underscores that DEP from different sources may induce inflammation through different mechanisms, triggered by different DEP-constituents. It should also be considered that even with a single-source DEP it seems unlikely, given the multitude of chemical components adhered to the particle surface, that there exists a simple mechanism explaining the cellular effects. Moreover, as the concentration-effect course of toxicity by different DEP-constituents most probably differs, the complexity of DEP-induced toxicity is also likely to increase by increasing concentrations, as more and more DEP-constituents enter toxic levels. As a consequence, mechanisms of effects observed at high DEP-concentrations in *in vitro* studies may not necessarily be directly relevant for the effects of low-level DEP-concentrations in real-life exposure. 

As within most fields of particle toxicity, oxidative stress is considered a main mechanism of DEP-induced toxicity and inflammation [7, 88, 89]. DEP-induced ROS-formation may activate redox-sensitive transcription factors involved in regulation of pro-inflammatory genes, such as NF-*κ*B and Nrf2 [18, 19]. ROS may interfere with various cell signaling pathways by inhibition of phosphatases through binding/oxidation of important thiol groups subsequently leading to increased phosphorylation/activation of protein kinases. In line with this, genotype variation in antioxidant enzymes, such as glutathione-s-transferases (GSTs), has been associated with susceptibility towards DEP-induced allergic inflammation in humans [90]. However, since GSTs are phase II metabolizing enzymes, also involved in detoxification of organic chemicals present in DEP, it cannot be excluded that the protective role of GSTs in DEP-induced inflammation extends beyond ROS-scavenging.

Direct ROS formation by DEP may arise from enzymatic metabolism of organic compounds such as PAH [18, 91]. However, several studies show that DEP also exert oxidative effects in acellular model systems. In a study of DEP produced by 4 different engine technologies (Euro1 to Euro 4), the antioxidant (DTT) consumption as a measure of oxidative capacity showed a correlation with the content of elemental carbon, water-insoluble carbon, and organic carbon [43]. Moreover, the two different reference-DEP, NIST 1650 and 2975, have been found to exhibit different oxidative capacity in cell free systems, possibly due to difference in chemical composition [16]. Furthermore, Mudway and colleagues [92] showed that DEP depleted lung lining fluid antioxidant levels *in vitro*. In contrast, a comparable effect of DEP was not observed in the airways of healthy subjects; and in a cell culture model *in vitro* the ratio of oxidised to nonoxidised glutathione did not change significantly after exposure to two different diesels, DEPa and NIST 2975 [30]. Thus, possibly the antioxidant defence in the lung lining fluid [92] and lung epithelial cells of healthy individuals are capable of dealing with the oxidative challenge posed by DEP at environmentally relevant concentrations. In a recent study of DEP from different fuel types, produced in the presence or absence of particle filter technology (PDF-treatment), we did not observe any correlation between a cellular DEP-induced ROS formation and DEP-induced cytokine responses in human bronchial epithelial BEAS-2B cells (Gerlofs-Nijland et al. unpublished results). This observation resembles previous results of studies on mineral particles showing no clear correlation between acellular ROS-formation and pro-inflammatory responses in *in vitro* cell culture models [93, 94]. 

DEP may also stimulate cellular generation of ROS as well as reactive nitrogen species (RNS) through activation of nitric oxide synthetase (iNOS) [29, 95, 96]. Moreover, DEP-induced MMP-1 responses in human alveolar type-2 like A549 cells appeared to be dependent on activation of cellular ROS-formation by the NADPH-oxidase analogue NOX4 [29]. NADPH-oxidase-mediated ROS formation also seems to regulate DEP-induced TNF-*α* responses in isolated rat brain capillaries [97], and toxicity of DEP in dopaminergic neurons [98]. Moreover, the DEP-component 1,2-naphthoquinone (1,2-NQ) was reported to induce IL-8 responses in human bronchial epithelial BEAS-2B cells, through mitochondrial H_2_O_2_-production [99]. Thus, it is conceivable that much of the reported suppressive effects of antioxidants on DEP-induced pro-inflammatory responses may be due to interference with DEP-induced cellular ROS-generation, rather than the direct particle-derived ROS production observed in acellular systems. If this is the case, oxidative stress should be considered a cellular response to DEP exposure and not a direct DEP property. However, findings obtained by use of antioxidants need to be interpreted with caution. While antioxidants may attenuate DEP-induced inflammation, the role of oxidative stress in cellular responses is inherently difficult to interpret. Antioxidants like N-acetyl cysteine (NAC) may also detoxify other reactive electrophilic DEP-constituents that potentially could trigger inflammatory reactions. Furthermore, ROS is an important and natural second messenger in most signaling pathways [100–102]. Thus, use of antioxidants is likely to interfere with a variety of cellular responses irrespective of oxidative stress. Few, if any, studies using antioxidants to assess the role of oxidative stress in particle induced effects have included proper controls to clarify these issues. It has also been pointed out that *in vitro* ROS-formation may have limited value in predicting pathological effects, because almost all particles elicit oxidative stress in cells, given a sufficient concentration [103]. Finally, oxidative stress alone may not be sufficient to induce pro-inflammatory responses in lung cells [104, 105], thus other and/or additional mechanisms are likely involved in DEP-induced inflammation. 

Of particular interest, recent studies show that pre-year-2000 DEP may induce Ca^2+^-signaling through activation of transient receptor potential (TRP) cation-channels in primary and transformed human bronchial epithelial cells (NHBE and BEAS-2B cells). Li and colleagues [106] have shown that DEP triggered Ca^2+^-influx through proteinase-activated receptor-2 (PAR-2) mediated activation of TRPV4 channels on the surface of human bronchial epithelial cells leading to increased expression of matrix metalloproteinase-1 (MMP-1). Ca^2+^-signaling appears to be central to IL-8 responses in bronchial epithelial cells induced by multiple compounds found in ambient air [107]. Therefore, it seems likely that DEP-induced PAR-2/TRPV4-activation is not restricted to MMP-1 regulation, but also involved in cytokine responses. In support of this, recent results from our lab show that silencing of PAR-2 by siRNA attenuated DEP-induced IL-6 responses in bronchial epithelial BEAS-2B cells (Øvrevik et al., unpublished results). Whether DEP activates PAR-2 receptors directly remains to be clarified. However, in line with previous studies on DEP-induced inflammation the effects appeared to be due to the soluble organic fraction of the particles [106]. These authors also showed that a TRPV4 polymorphism (TRPV4_P19S_) associated with increased susceptibility to COPD significantly enhanced DEP-induced Ca^2+^-signaling and MMP-1 responses, thus providing a possible link between COPD pathogenesis and DEP-exposure. In parallel to these observations, Deering-Rice and co-workers [27] found that DEP (black smoker, Table 1) induced Ca^2+^-signaling by activating TRPA1 receptors in sensory nerve cells. This effect was attributed to electrophilic components of DEP, including various aldehydes and quinones [27]. In further support of these findings, the DEP-component 1,2-NQ has been found to activate TRPV1 (vanilloid receptor-1) in guinea pig trachea [108]. However, this study suggests that TRPV1 was indirectly activated by 1,2-NQ through transactivation of protein tyrosine kinases such as the epidermal growth factor receptor (EGFR).

It is interesting to note that cellular signaling through RAGE (receptors for advanced glycation end-products) was suggested to have a role in DEP-induced NF-*κ*B-activation and chemokine responses (MCP-1 and CINC-1) in a type-I-like epithelial cell line (R3/1) [109]. In line with this, DEP-exposure also enhanced RAGE-expression in R3/1 cells and primary human small airway epithelial cells (SAECs), possibly providing a positive feedback mechanism for DEP-induced inflammation [109]. However, as with the studies on PAR-2 and TRP-channels, it is still unclear whether DEP activated RAGE directly or whether DEP or DEP-components caused formation of RAGE-ligands in the exposed cells. 

Studies also suggest that DEP exposure activates EGFR-signaling [24, 107, 110, 111]. Of notice, the EGFR does not seem to be a direct target of DEP or DEP-components, but is more likely a downstream response to some DEP-triggered effect. Activation of EGFR-signaling through cleavage and release of membrane bound transforming growth factor (TGF-*α*) by the metalloproteinase TNF-*α* converting enzyme (TACE or ADAM17) seems to be a universal mechanisms of IL-8 regulation in airway epithelial cells by multiple endogenous and exogenous compounds, including DEP and various air pollution components [107, 112, 113]. In coherence with reported *in vitro* effects increased EGFR-expression and activation have been observed in biopsies of bronchial epithelium from volunteers exposed to freshly generated DEP [114]. Moreover, TACE and EGFR are overexpressed in pulmonary epithelium of asthmatics and COPD patients and this correlates with increased expression of IL-8, which is a key activator of neutrophils [115]. Thus, increased TACE and EGFR-expression may be important susceptibility factors for neutrophilic inflammation by air pollutants. 

Considerable progress has been made to elucidate the mechanisms of DEP-induced inflammation, beyond the mere oxidative stress effects. Whether any of the above mentioned receptors are direct targets of DEP or organic chemicals from DEP remains to be clarified. However, depending on the further research, these receptors may turn out as important susceptibility factors for adverse effects of DEP-exposure. Such knowledge may also substantiate any possible role of biomarkers of effect as measured by gene-array. Another important aspect to consider is that if DEP-induced pro-inflammatory responses are regulated by different cell surface receptors, this is likely to give rise to cell specific effects, since receptor expression may be highly cell-type dependent. Similarly, expression of metabolizing enzymes involved in bioactivation or ROS-formation from adhered hydrocarbons may also vary between different cell-types and affect the outcome of exposure. It is conceivable that this may explain apparent discrepancies in effects obtained by different *in vitro* models, such as the reported biodiesel effects discussed above. For the same reason, care should be taken when interpreting the importance of results obtained by a single cell model. 

## 6. Challenges and Concluding Remarks 

Diesel engine exhaust represents a complex and variable air pollution mixture, of which the physicochemical characteristics are highly dependent on the fuel used and the type of engine [43, 116]. Recently, Hesterberg and colleagues have stressed this important issue, by questioning the relevance of certain samples or exhaust exposures that currently are used in experimental studies, for risk-assessment of particulate matter from new technology diesel exhaust [74]. As shown in Table 1, a large proportion of the *in vitro* studies have used the standard reference diesel material from the National Institute of Standards and Technology (NIST, USA), which were collected from a fork lift truck several years ago. Although a thoroughly characterised material may be very useful for investigating the role of the physicochemical composition for the effects, one may question whether it is time to produce and agree on a commercial reference diesel sample which is more representative to current diesel emissions.

*In vitro *studies represent a fast and convenient system which may have implications for technology development. *In vitro* studies are also of key importance for increasing our knowledge about the underlying biological mechanisms of effects. Interestingly, several of the proteins investigated in *in vitro* studies of mechanisms of signal transduction, have also been observed activated in bronchial biopsies from human volunteers exposed to diesel exhaust particles in clinical studies [15, 114, 117, 118]. Thus there is in many cases a coherence of *in vitro* and *in vivo *findings. 

With respect to *in vitro* research on the pulmonary effects of diesel engine exhaust emissions on inflammatory reactions, a large diversity of *in vitro* models has been applied, and a range of effect parameters have been investigated. In addition, several different types of DEP samples have been used, subjected to different treatments for exposure. The various models have their advantages and disadvantages, but the diversity can be seen as strength, though it challenges the process of generating overall conclusions. Furthermore, for a complete evaluation of DEP effects also genotoxic and other outcomes should be taken into consideration.

A well-known limitation of *in vitro* studies is the general use of exposure concentrations that are on the high side, compared to real world situations. The exact levels to which pulmonary cells are likely to be exposed to *in vivo* are difficult to estimate, based on the complexity of the deposition pattern. However, according to estimations of Li and colleagues, a biologically relevant tissue culture concentration of DEP ranges from 0.2 to 20 ug/cm^2^ [119]. Although the concentrations used in several studies fall within this range, and certain effects also have been detected at concentrations below this range, it would be important to optimize and increase the sensitivity of current *in vitro* models. Of notice, direct exposure to freshly generated DEP of cells at the air/liquid interface (ALI) has been performed [42, 120–122]. Results reported from these rather complicated models support results obtained by exposing traditional submerged cell cultures with DEP collected on filters. This may be due to similar amounts of organic components being released independently of the aggregation/agglomeration state. However, some studies suggest that response to ALI exposure may occur at lower DEP-doses that by conventional exposure of submerged cell cultures [120–122]. These are important observations considering that *in vitro* studies often are criticized for using too high particle concentrations.

The inflammatory effects of DEP seem to be attributable to the soluble organic fraction, but questions still remain with respect to what fraction and components that are most important for the inflammatory responses. The mechanisms are still unclear, but receptors in the plasma membrane, including the PAR-2 receptor, vanilloid-1 receptor, RAGE-receptor, and the EGF-receptor, seem to be involved. DEP are known to induce acellular as well as cell-mediated ROS-formation, oxidative stress and deplete the levels of antioxidants, which seem to be involved in the inflammatory effects of DEP. At what stage the oxidative tonus exerts its major effect(s) in the signalling pathways leading to inflammation remains to be further clarified.

Of notice, engine and fuel technology have been rapidly changing resulting in reduced emissions. The question arises whether this reduction in DEP from modern engines has resulted in an equivalent reduction in harmful properties of the emissions. Moreover, increased use of biodiesel to meet demands for CO_2_-neutral fuels warrants further studies on how different fuels affect the pro-inflammatory properties of DEP. 

## Figures and Tables

**Table 1:** Origin of diesel particles used in the different studies, test systems, concentrations and effects on different end-points. The arrows give a rough indication of the magnitude of effects judged from the results presented (small: ↑; moderate: ↑↑; strong ↑↑↑).

Diesel type	Test system	Concentrations	Endpoints	Citation
Heavy duty machine NIST 1650	Cell-free	Not relevant	ROS (malondialdehyde) ↑↑	Ball et al. [16]
Heavy duty machine NIST 1650	Bronchial epithelial cells (16HBE cell line), Primary human nasal epithelial cells	10–30 *μ*g/cm^2^	ROS-formation (DCF-fluoroscence) ↑↑, CYPIAI mRNA and EROD-activity ↑↑; NADPH quinone oxidodreductase-1 mRNA ↑↑; translocation of transcription factor Nrf2 ↑↑	Baulig et al., [17, 18]
Heavy duty machine NIST 1650	16-HBE (bronchial epithelial cells)	10 *μ*g/cm^2^	GM-CSF ↑↑, NF*κ*-B activity ↑↑, CYP1A1 mRNA ↑↑, Phosphorylation of MAPK Erk and p38 ↑↑	Bonvallot et al., [19]
Heavy duty machine NIST 1650	THP-1 monocyte and A549 epithelial cell co-culture	10–40 *μ*g/cm^2^	IL-6 ↑; IL-8 ↑; TNF*α*	Kocbach et al., [20]
Heavy duty machine NIST 1650	A549 epithelial cells	0.1 to 20 ppm	IL-8 ↑; CRP ↑	Patel et al., [21]
Heavy duty machine NIST 1650	RAW monocyte/macrophages	5–20 *μ*g/mL	NO-production ↑↑	Saxena et al., [22]
Heavy duty machine NIST 1650	BEAS-2B, (bronchial epithelial cells)	~4–60 *μ*g/cm^2^	Increased cytotoxicity	Totlandsdal et al., [23]
Forklift NIST 2975	Cell-free,	Not relevant	ROS (malondialdehyde) ↑	Ball et al. [16]
Forklift NIST 2975	Primary human epithelial cells	50 *μ*g/mL	Phosphorylation of Stat3, EGF-receptor ↑↑	Cao et al., [24]
Forklift NIST 2975	BEAS-2B Bronchial epithelial cells	10 *μ*g/cm^2^	STAT3; src; EGFR necessary for p21 ↑↑; inhibition of proliferation	Cao et al., [25]
Forklift NIST 2975	BEAS-2B Bronchial epithelial cells + primary monocytes	50 *μ*g/mL	IL-1	Chaudhuri et al., [26]
Forklift NIST 2975	HEK-293 epidermal cells, primary mouse neurons	77–770 *μ*g/mL	TRPA-1 activation ↑	Deering-Rice et al., [27]
Forklift NIST 2975	16-HBE, monocytes, dendritic cells, triple co-culture	125 *μ*g/mL (cells on insert)	Reduction and altered distribution of occluding, minor effect in epithelial cells, stronger in other cell types	Lehmann et al., [28]
Forklift NIST 2975	A549 and NCI-H292 cells	5–10 *μ*g/cm^2^	MMP-1	Amara et al., [29]

Forklift NIST 2975	Primary murine tracheal cells	25 *μ*g/cm^2^	Small effects on LDH, HO-1 Alveolar lung cell line GSH/GSSG ratio	Manzo et al., [30]
Forklift NIST 2975	Primary human macrophages Suspension culture,	100 *μ*g/mL	No significant increase in TNF or IL-8; DEP reduced cytokine release induced by LPS	Sawyer et al., [31]
Forklift NIST 2975	BEAS-2B (bronchial epithelial cells)	Only extracts tested	IL.6 ↑; IL-8 ↑	Swanson et al., [32]
Forklift NIST 2975	BEAS-2B cells (bronchial epithelial cells), HAEC cells	10 *μ*g/cm^2^	IL-8 mRNA ↑↑, N-DEP effect NF*κ*B dependent	Tal et al., [33]
NIST, not specified	A549 epithelial cells and primary rat airway epithelial cells	20 *μ*g/cm^2^	Transepithelial conductance ↑ Occluding ↓	Caraballo et al., [34]
C-DEP, EPA diesel (2005)	Primary human epithelial cells	50 *μ*g/mL	Phosphorylation of Stat3 ↑↑↑	Cao et al., [24]
A-DEP, diesel from Sagai et al., [35]	BEAS-2B (bronchial epithelial cells)	10 *μ*g/cm^2^	IL-8 mRNA ↑↑↑	Tal et al., [33]
A-DEP, diesel from Sagai et al., [35]	BEAS-2B (bronchial epithelial cells)	5–50 *μ*g/mL	HSP70 ↑↑ at 10 *μ*g/mL	Jung et al., [36]
2.7 L Isuzu diesel; A-DEP diesel from Sagai et al., [35]	BEAS-2B (bronchial epithelial cells) and primary peripheral airway cells	5 and 25 *μ*g/mL	IL-8 ↑↑	Kawasaki et al., [37]
2.7 L Isuzu diesel; A-DEP before year 2000	BEAS-2B (bronchial epithelial cells)	5–100 *μ*g/mL	IL-8 ↑↑, GM-CSF ↑↑, Depending on NF*κ*B, reduced by NAC	Takizawa et al., [38, 39]
2.7 L Isuzu diesel; A-DEP before year 2000	BEAS-2B (bronchial epithelial cells)	5–100 *μ*g/mL	IL-8 ↑↑, RANTES ↑↑; dependent on p38, reduced by NAC	Hashimoto et al., [40]
Deutz unloaded 2.2 L; EURO 4 (2009)	BEAS-2B (bronchial epithelial cells)	~4–60 *μ*g/cm^2^	IL-6 ↑↑; IL-8 ↑↑; CYP1A1 ↑↑↑ (at 0.004 *μ*g/cm^2^); COX-2 ↑↑↑; p38 ↑ and NF*κ*B ↑	Totlandsdal et al., [23]
DEP_A_ (from EPA)	Primary murine tracheal cells	5–200 *μ*g/cm^2^	LDH ↑ at 100 *μ*g/cm^2^ Alveolar lung cell line HO-1 ↑↑	Manzo et al., [30]
1.6 L Volkswagen diesel; from EPA (1992)	Primary human bronchial epithelial cells; human primary monocytes differentiated to dendritic cells;	3 *μ*g/cm^2^	Epithelial cells TSLP ↑↑ Dendritic cells OX40L ↑↑;	Bleck et al., [41]
Heavy duty 9.2 L; DEP freshly generated	A549 epithelial cells, Air-liquid interface exposure	Low 0.1 mg/m^3^ High 0.8 mg/m^3^	IL-1*β* only at one conc of high NO_2_, reduced viability	Tsukue et al., [42]
2.2 L Honda EURO 4 machine DEP + rape seed biodiesel (±DPF)	BEAS-2B (bronchial epithelial cells)	~6 to 200 *μ*g/mL	IL-6 Most effect in *μ*g/mL B50 DPF ≫ B0DPF > B50 > B0	Gerlofs-Nijland et al., submitted
2.2 L Honda EURO 4 machine, Golf. Corolla cars DPF, diesel biodiesel	Cell-free	Not relevant	Correlation of DTT consumption with EC/Water insoluble OC/OC	Ka et al., [43]
US 2004 machine (black smoker)	HEK-293 epidermal cells, primary mouse neurons	77–770 *μ*g/mL	TRPA-1 activation ↑↑	Deering-Rice et al., [27]
Soy bean biodiesel 2005	BEAS-2B (bronchial epithelial cells)	Only extracts tested	Stronger effects of biodiesel (soy bean)	Swanson et al., [32]

## References

[1] WHO (2005). *Health Effects of Transport-Related Air Pollution*.

[2] van Kempen E, Babisch W (2012). The quantitative relationship between road traffic noise and hypertension: a meta-analysis. *Journal of Hypertension*.

[3] Cohen AJ, Anderson HR, Ostro B (2005). The global burden of disease due to outdoor air pollution. *Journal of Toxicology and Environmental Health*.

[4] Anderson JO, Thundiyil JG, Stolbach A (2012). Clearing the air: a review of the effects of particulate matter air pollution on human health. *Journal of Medical Toxicology*.

[5] Brook RD, Rajagopalan S, Pope CA (2010). Particulate matter air pollution and cardiovascular disease: an update to the scientific statement from the American Heart Association. *Circulation*.

[6] Salvi S, Holgate ST (1999). Mechanisms of particulate matter toxicity. *Clinical and Experimental Allergy*.

[7] Donaldson K, Stone V (2003). Current hypotheses on the mechanisms of toxicity of ultrafine particles. *Annali dell’Istituto Superiore di Sanita*.

[8] Kelly FJ, Fussell JC (2011). Air pollution and airway disease. *Clinical and Experimental Allergy*.

[9] Ristovski ZD, Miljevic B, Surawski NC (2012). Respiratory health effects of diesel particulate matter. *Respirology*.

[10] Emmendoerffer A, Hecht M, Boeker T, Mueller M, Heinrich U (2000). Role of inflammation in chemical-induced lung cancer. *Toxicology Letters*.

[11] Bartsch H, Nair J (2006). Chronic inflammation and oxidative stress in the genesis and perpetuation of cancer: role of lipid peroxidation, DNA damage, and repair. *Langenbeck’s Archives of Surgery*.

[12] Donaldson K, Stone V, Seaton A, MacNee W (2001). Ambient particle inhalation and the cardiovascular system: potential mechanisms. *Environmental Health Perspectives*.

[13] Frampton MW (2001). Systemic and cardiovascular effects of airway injury and inflammation: ultrafine particle exposure in humans. *Environmental Health Perspectives*.

[14] Gan WQ, Man SFP, Senthilselvan A, Sin DD (2004). Association between chronic obstructive pulmonary disease and systemic inflammation: a systematic review and a meta-analysis. *Thorax*.

[15] Pourazar J, Mudway IS, Samet JM (2005). Diesel exhaust activates redox-sensitive transcription factors and kinases in human airways. *American Journal of Physiology*.

[16] Ball JC, Straccia AM, Young WC, Aust AE (2000). The formation of reactive oxygen species catalyzed by neutral, aqueous extracts of NIST ambient particulate matter and diesel engine particles. *Journal of the Air and Waste Management Association*.

[17] Baulig A, Sourdeval M, Meyer M, Marano F, Baeza-Squiban A (2003). Biological effects of atmospheric particles on human bronchial epithelial cells. Comparison with diesel exhaust particles. *Toxicology in Vitro*.

[18] Baulig A, Garlatti M, Bonvallot V (2003). Involvement of reactive oxygen species in the metabolic pathways triggered by diesel exhaust particles in human airway epithelial cells. *American Journal of Physiology*.

[19] Bonvallot V, Baeza-Squiban A, Baulig A (2001). Organic compounds from diesel exhaust particles elicit a proinflammatory response in human airway epithelial cells and induce cytochrome p450 1A1 expression. *American Journal of Respiratory Cell and Molecular Biology*.

[20] Kocbach A, Herseth JI, Låg M, Refsnes M, Schwarze PE (2008). Particles from wood smoke and traffic induce differential pro-inflammatory response patterns in co-cultures. *Toxicology and Applied Pharmacology*.

[21] Patel H, Eo S, Kwon S (2011). Effects of diesel particulate matters on inflammatory responses in static and dynamic culture of human alveolar epithelial cells. *Toxicology Letters*.

[22] Saxena QB, Saxena RK, Siegel PD, Lewis DM (2003). Identification of organic fractions of diesel exhaust particulate (DEP) which inhibit nitric oxide (NO) production from a murine macrophage cell line. *Toxicology Letters*.

[23] Totlandsdal AI, Cassee FR, Schwarze P, Refsnes M, Låg M (2010). Diesel exhaust particles induce CYP1A1 and pro-inflammatory responses via differential pathways in human bronchial epithelial cells. *Particle and Fibre Toxicology*.

[24] Cao D, Bromberg PA, Samet JM (2007). COX-2 expression induced by diesel particles involves chromatin modification and degradation of HDAC1. *American Journal of Respiratory Cell and Molecular Biology*.

[25] Cao D, Bromberg PA, Samet JM (2010). Diesel particle-induced transcriptional expression of p21 involves activation of EGFR, Src, and Stat3. *American Journal Respiratory Cell Molecular Biology*.

[26] Chaudhuri N, Paiva C, Donaldson K, Duffin R, Parker LC, Sabroe I (2010). Diesel exhaust particles override natural injury-limiting pathways in the lung. *American Journal of Physiology*.

[27] Deering-Rice CE, Romero EG, Shapiro D (2011). Electrophilic components of diesel exhaust particles (DEP) activate transient receptor potential ankyrin-1 (TRPA1): a probable mechanism of acute pulmonary toxicity for DEP. *Chemical Research in Toxicology*.

[28] Lehmann AD, Blank F, Baum O, M PGehrB (2009). Rothen-Rutishauser: diesel exhaust particles modulate the tight junction protein occludin in lung cells in vitro. *Particle and Fibre Toxicology*.

[29] Amara N, Bachoual R, Desmard M (2007). Diesel exhaust particles induce matrix metalloprotease-1 in human lung epithelial cells via a NADP(H) oxidase/NOX4 redox-dependent mechanism. *American Journal of Physiology*.

[30] Manzo ND, Slade R, Richards JH, McGee JK, Martin LD, Dye JA (2010). Susceptibility of inflamed alveolar and airway epithelial cells to injury induced by diesel exhaust particles of varying organic carbon content. *Journal of Toxicology and Environmental Health*.

[31] Sawyer K, Mundandhara S, Ghio AJ, Madden MC (2010). The effects of ambient particulate matter on human alveolar macrophage oxidative and inflammatory responses?. *Journal of Toxicology and Environmental Health Part A*.

[32] Swanson KJ, Kado NY, Funk WE, Pleil JD, Madden MC, Ghio AJ (2009). Release of the pro-inflammatory markers by BEAS-2B cells following in vitro exposure to biodiesel extracts. *Open Toxicology Journal*.

[33] Tal TL, Simmons SO, Silbajoris R (2010). Differential transcriptional regulation of IL-8 expression by human airway epithelial cells exposed to diesel exhaust particles. *Toxicology and Applied Pharmacology*.

[34] Caraballo JC, Yshii C, Westphal W, Moninger T, Comellas AP (2011). Ambient particulate matter affects occludin distribution and increases alveolar transepithelial electrical conductance. *Respirology*.

[35] Sagai M, Saito H, Ichinose T, Kodama M, Mori Y (1993). Biological effects of diesel exhaust particles. I. In vitro production of superoxide and in vivo toxicity in mouse. *Free Radical Biology and Medicine*.

[36] Jung EJ, Avliyakulov NK, Boontheung P, Loo JA, Nel AE (2007). Pro-oxidative DEP chemicals induce heat shock proteins and an unfolding protein response in a bronchial epithelial cell line as determined by DIGE analysis. *Proteomics*.

[37] Kawasaki S, Takizawa H, Takami K (2001). Benzene-extracted components are important for the major activity of diesel exhaust particles: effect on interleukin-8 gene expression in human bronchial epithelial cells. *American Journal of Respiratory Cell and Molecular Biology*.

[38] Takizawa H, Ohtoshi T, Kawasaki S (2000). Diesel exhaust particles activate human bronchial epithelial cells to express inflammatory mediators in the airways: a review. *Respirology*.

[39] Takizawa H, Abe S, Ohtoshi T (2000). Diesel exhaust particles up-regulate expression of intercellular adhesion molecule-1 (ICAM-1) in human bronchial epithelial cells. *Clinical and Experimental Immunology*.

[40] Hashimoto S, Gon Y, Takeshita I (2000). Diesel exhaust particles activate p38 MAP kinase to produce interleukin 8 and RANTES by human bronchial epithelial cells and N-acetylcysteine attenuates p38 MAP kinase activation. *American Journal of Respiratory and Critical Care Medicine*.

[41] Bleck B, Tse DB, Curotto De Lafaille MA, Zhang F, Reibman J (2008). Diesel exhaust particle-exposed human bronchial epithelial cells induce dendritic cell maturation and polarization via thymic stromal lymphopoietin. *Journal of Clinical Immunology*.

[42] Tsukue N, Okumura H, Ito T, Sugiyama G, Nakajima T (2010). Toxicological evaluation of diesel emissions on A549 cells. *Toxicology in Vitro*.

[43] Ka LC, Polidori A, Ntziachristos L (2009). Chemical characteristics and oxidative potential of particulate matter emissions from gasoline, diesel, and biodiesel cars. *Environmental Science and Technology*.

[44] Davies DE, Holgate ST (2002). Asthma: the importance of epithelial mesenchymal communication in pathogenesis: inflammation and the airway epithelium in asthma. *International Journal of Biochemistry and Cell Biology*.

[45] Bogaert P, Tournoy KG, Naessens T, Grooten J (2009). Where asthma and hypersensitivity pneumonitis meet and differ: noneosinophilic severe asthma. *American Journal of Pathology*.

[46] Sutherland ER, Martin RJ (2003). Airway inflammation in chronic obstructive pulmonary disease: comparisons with asthma. *Journal of Allergy and Clinical Immunology*.

[47] Oo YH, Adams DH (2010). The role of chemokines in the recruitment of lymphocytes to the liver. *Journal of Autoimmunity*.

[48] Gross O, Thomas CJ, Guarda G, Tschopp J (2011). The inflammasome: an integrated view. *Immunological Reviews*.

[49] Pétrilli V, Dostert C, Muruve DA, Tschopp J (2007). The inflammasome: a danger sensing complex triggering innate immunity. *Current Opinion in Immunology*.

[50] Provoost S, Maes T, Pauwels NS (2011). NLRP3/caspase-1-independent IL-1beta production mediates diesel exhaust particle-induced pulmonary inflammation. *Journal of Immunology*.

[51] Gooz M (2010). ADAM-17: the enzyme that does it all. *Critical Reviews in Biochemistry and Molecular Biology*.

[52] Shaw CA, Robertson S, Miller MR (2011). Diesel exhaust particulate—exposed macrophages cause marked endothelial cell activation. *American Journal of Respiratory Cell and Molecular Biology*.

[53] Porter M, Karp M, Killedar S (2007). Diesel-enriched particulate matter functionally activates human dendritic cells. *American Journal of Respiratory Cell and Molecular Biology*.

[54] Yang HM, Ma JYC, Castranova V, Ma JKH (1997). Effects of diesel exhaust particles on the release of interleukin-1 and tumor necrosis factor-alpha from rat alveolar macrophages. *Experimental Lung Research*.

[55] Dong W, Lewtas J, Luster MI (1996). Role of endotoxin in tumor necrosis factor *α* expression from alveolar macrophages treated with urban air particles. *Experimental Lung Research*.

[56] Amakawa K, Terashima T, Matsuzaki T, Matsumaru A, Sagai M, Yamaguchi K (2003). Suppressive effects of diesel exhaust particles on cytokine release from human and murine alveolar macrophages. *Experimental Lung Research*.

[57] Song JJ, Lee JD, Lee BD, Chae SW, Park MK (2012). Effect of diesel exhaust particles on human middle ear epithelial cells. *International Journal of Pediatric Otorhinolaryngology*.

[58] Ushio H, Nohara K, Fujimaki H (1999). Effect of environmental pollutants on the production of pro-inflammatory cytokines by normal human dermal keratinocytes. *Toxicology Letters*.

[59] Saber AT, Bornholdt J, Dybdahl M (2005). Tumor necrosis factor is not required for particle-induced genotoxicity and pulmonary inflammation. *Archives of Toxicology*.

[60] Schwarze PE, Totlandsdal AI, Herseth JI, Rijeka VV (2010). Importance of sources and components of particulate air pollution for cardio-pulmonary inflammatory responses. *Air Pollution*.

[61] Ziegler SF, Artis D (2010). Sensing the outside world: TSLP regulates barrier immunity. *Nature Immunology*.

[62] Bleck B, Tse DB, Gordon T, Ahsan MR, Reibman J (2010). Diesel exhaust particle-treated human bronchial epithelial cells upregulate Jagged-1 and OX40 ligand in myeloid dendritic cells via thymic stromal lymphopoietin. *Journal of Immunology*.

[63] Provoost S, Maes T, Willart MAM, Joos GF, Lambrecht BN, Tournoy KG (2010). Diesel exhaust particles stimulate adaptive immunity by acting on pulmonary dendritic cells. *Journal of Immunology*.

[64] Totlandsdal AI, Refsnes M, Skomedal T, Osnes JB, Schwarze PE, Låg M (2008). Particle-induced cytokine responses in cardiac cell cultures—the effect of particles versus soluble mediators released by particle-exposed lung cells. *Toxicological Sciences*.

[65] Hofer TPJ, Bitterle E, Beck-Speier I (2004). Diesel exhaust particles increase LPS-stimulated COX-2 expression and PGE_2_ production in human monocytes. *Journal of Leukocyte Biology*.

[66] Gualtieri M, Øvrevik J, Mollerup S (2011). Airborne urban particles (Milan winter-PM2.5) cause mitotic arrest and cell death: effects on DNA, mitochondria, AhR binding and spindle organization. *Mutation Research*.

[67] Li N, Venkatesan MI, Miguel A (2000). Induction of heme oxygenase-1 expression in macrophages by diesel exhaust particle chemicals and quinones via the antioxidant-responsive element. *Journal of Immunology*.

[68] Li N, Alam J, Venkatesan MI (2004). Nrf2 is a key transcription factor that regulates antioxidant defense in macrophages and epithelial cells: protecting against the proinflammatory and oxidizing effects of diesel exhaust chemicals. *Journal of Immunology*.

[69] Tian Y, Rabson AB, Gallo MA (2002). Ah receptor and NF-*κ*B interactions: mechanisms and physiological implications. *Chemico-Biological Interactions*.

[70] Zordoky BNM, El-Kadi AOS (2009). Role of NF-*κ*B in the regulation of cytochrome P450 enzymes. *Current Drug Metabolism*.

[71] Monteleone I, MacDonald TT, Pallone F, Monteleone G (2012). The aryl hydrocarbon receptor in inflammatory bowel disease: linking the environment to disease pathogenesis. *Current Opinion in Gastroenterology*.

[72] Ritz SA, Wan J, Diaz-Sanchez D (2007). Sulforaphane-stimulated phase II enzyme induction inhibits cytokine production by airway epithelial cells stimulated with diesel extract. *American Journal of Physiology*.

[73] Matti Maricq M (2007). Chemical characterization of particulate emissions from diesel engines: a review. *Journal of Aerosol Science*.

[74] Hesterberg TW, Long CM, Sax SN (2011). Particulate matter in new technology diesel exhaust (NTDE) is quantitatively and qualitatively very different from that found in traditional diesel exhaust (TDE). *Journal of the Air & Waste Management Association*.

[75] Ma JYC, Ma JKH (2002). The dual effect of the particulate and organic components of diesel exhaust particles on the alteration of pulmonary immune/inflammatory responses and metabolic enzymes. *Journal of Environmental Science and Health*.

[76] Siegel PD, Saxena RK, Saxena QB (2004). Effect of diesel exhaust particulate (DEP) on immune responses: contributions of particulate versus organic soluble components. *Journal of Toxicology and Environmental Health*.

[77] Takano H, Yanagisawa R, Inoue KI (2007). Components of diesel exhaust particles diversely enhance a variety of respiratory diseases related to infection or allergy: extracted organic chemicals and the residual particles after extraction differently affect respiratory diseases. *Journal of Clinical Biochemistry and Nutrition*.

[78] Totlandsdal AI, Herseth JI, Bolling AK (2012). Differential effects of the particle core and organic extract of diesel exhaust particles. *Toxicology Letters*.

[79] Holder AL, Lucas D, Goth-Goldstein R, Koshland CP (2007). Inflammatory response of lung cells exposed to whole, filtered, and hydrocarbon denuded diesel exhaust. *Chemosphere*.

[80] Fuentes-Mattei E, Rivera E, Gioda A, Sanchez-Rivera D, Roman-Velazquez FR, Jimenez-Velez BD (2010). Use of human bronchial epithelial cells (BEAS-2B) to study immunological markers resulting from exposure to PM2.5 organic extract from Puerto Rico. *Toxicology and Applied Pharmacology*.

[81] Yanagisawa R, Takano H, Inoue KI (2006). Components of diesel exhaust particles differentially affect Th1/Th2 response in a murine model of allergic airway inflammation. *Clinical and Experimental Allergy*.

[82] Swanson KJ, Madden MC, Ghio AJ (2007). Biodiesel exhaust: the need for health effects research. *Environmental Health Perspectives*.

[83] Brito JM, Belotti L, Toledo AC (2010). Acute cardiovascular and inflammatory toxicity induced by inhalation of diesel and biodiesel exhaust particles. *Toxicological Sciences*.

[84] Jalava PI, Tapanainen M, Kuuspalo K (2010). Toxicological effects of emission particles from fossil- and biodiesel-fueled diesel engine with and without DOC/POC catalytic converter. *Inhalation Toxicology*.

[85] Hemmingsen JG, Moller P, Nojgaard JK, Roursgaard M, Loft S (2011). Oxidative stress, genotoxicity, and vascular cell adhesion molecule expression in cells exposed to particulate matter from combustion of conventional diesel and methyl ester biodiesel blends. *Environmental Science & Technology*.

[86] Hayakawa K, Nakamura A, Terai N, Kizu R, Ando K (1997). Nitroarene concentrations and direct-acting mutagenicity of diesel exhaust particulates fractionated by silica-gel column chromatography. *Chemical and Pharmaceutical Bulletin*.

[87] Salmeen I, Durisin AM, Prater TJ (1982). Contribution of 1-nitropyrene to direct-acting Ames assay mutagenicities of diesel particulate extracts. *Mutation Research*.

[88] Schins RPF (2002). Mechanisms of genotoxicity of particles and fibers. *Inhalation Toxicology*.

[89] Li YJ, Takizawa H, Kawada T (2010). Role of oxidative stresses induced by diesel exhaust particles in airway inflammation, allergy and asthma: their potential as a target of chemoprevention. *Inflammation and Allergy*.

[90] Gilliland FD, Li YF, Saxon A, Diaz-Sanchez D (2004). Effect of glutathione-S-transferase M1 and P1 genotypes on xenobiotic enhancement of allergic responses: randomised, placebo-controlled crossover study. *The Lancet*.

[91] Yin XJ, Ma JYC, Antonini JM, Castranova V, Ma JKH (2004). Roles of reactive oxygen species and heme oxygenase-1 in modulation of alveolar macrophage-mediated pulmonary immune responses to *Listeria monocytogenes* by diesel exhaust particles. *Toxicological Sciences*.

[92] Mudway IS, Stenfors N, Duggan ST (2004). An in vitro and in vivo investigation of the effects of diesel exhaust on human airway lining fluid antioxidants. *Archives of Biochemistry and Biophysics*.

[93] Øvrevik J, Hetland RB, Schins RP, Myran T, Schwarze PE (2006). Iron release and ROS generation from mineral particles are not related to cytokine release or apoptosis in exposed A549 cells. *Toxicology Letters*.

[94] Clouter A, Brown D, Höhr D, Borm P, Donaldson K (2001). Inflammatory effects of respirable quartz collected in workplaces versus standard DQ12 quartz: particle surface correlates. *Toxicological Sciences*.

[95] Zhao H, Barger MW, Ma JKH, Castranova V, Ma JYC (2006). Cooperation of the inducible nitric oxide synthase and cytochrome P450 1A1 in mediating lung inflammation and mutagenicity induced by diesel exhaust particles. *Environmental Health Perspectives*.

[96] Zhao Y, Usatyuk PV, Gorshkova IA (2009). Regulation of COX-2 expression and IL-6 release by particulate matter in airway epithelial cells. *American Journal of Respiratory Cell and Molecular Biology*.

[97] Hartz AMS, Bauer B, Block ML, Hong JS, Miller DS (2008). Diesel exhaust particles induce oxidative stress, proinflammatory signaling, and P-glycoprotein up-regulation at the blood-brain barrier. *The FASEB Journal*.

[98] Block ML, Wu X, Pei Z (2004). Nanometer size diesel exhaust particles are selectively toxic to dopaminergic neurons: the role of microglia, phagocytosis, and NADPH oxidase. *The FASEB Journal*.

[99] Cheng WY, Currier J, Bromberg PA, Silbajoris R, Simmons SO, Samet JM (2012). Linking oxidative events to inflammatory and adaptive gene expression induced by exposure to an organic particulate matter component. *Environmental Health Perspectives*.

[100] Sauer H, Wartenberg M, Hescheler J (2001). Reactive oxygen species as intracellular messengers during cell growth and differentiation. *Cellular Physiology and Biochemistry*.

[101] Torres M, Forman HJ (2003). Redox signaling and the MAP kinase pathways. *BioFactors*.

[102] Guzik TJ, Korbut R, Adamek-Guzik T (2003). Nitric oxide and superoxide in inflammation and immune regulation. *Journal of Physiology and Pharmacology*.

[103] Donaldson K, Borm PJA, Castranova V, Gulumian M (2009). The limits of testing particle-mediated oxidative stress in vitro in predicting diverse pathologies; relevance for testing of nanoparticles. *Particle and Fibre Toxicology*.

[104] Oslund KL, Miller LA, Usachenko JL, Tyler NK, Wu R, Hyde DM (2004). Oxidant-injured airway epithelial cells upregulate thioredoxin but do not produce interleukin-8. *American Journal of Respiratory Cell and Molecular Biology*.

[105] Øvrevik J, Refsnes M, Schwarze P, Låg M (2008). The ability of oxidative stress to mimic quartz-induced chemokine responses is lung cell line-dependent. *Toxicology Letters*.

[106] Li J, Kanju P, Patterson M (2011). TRPV4-mediated calcium influx into human bronchial epithelia upon exposure to diesel exhaust particles. *Environmental Health Perspectives*.

[107] Ovrevik J, Refsnes M, Totlandsdal AI, Holme JA, Schwarze PE, Lag M (2011). TACE/TGF-alpha/EGFR regulates CXCL8 in bronchial epithelial cells exposed to particulate matter components. *European Respiratory Journal*.

[108] Kikuno S, Taguchi K, Iwamoto N (2006). 1,2-Naphthoquinone activates vanilloid receptor 1 through increased protein tyrosine phosphorylation, leading to contraction of guinea pig trachea. *Toxicology and Applied Pharmacology*.

[109] Reynolds PR, Wasley KM, Allison CH (2011). Diesel particulate matter induces receptor for advanced glycation end-products (RAGE) expression in pulmonary epithelial cells, and RAGE signaling influences NF-*κ*B-mediated inflammation. *Environmental Health Perspectives*.

[110] Blanchet S, Ramgolam K, Baulig A, Marano F, Baeza-Squiban A (2004). Fine particulate matter induces amphiregulin secretion by bronchial epithelial cells. *American Journal of Respiratory Cell and Molecular Biology*.

[111] Tal TL, Bromberg PA, Kim Y, Samet JM (2008). Epidermal growth factor receptor activation by diesel particles is mediated by tyrosine phosphatase inhibition. *Toxicology and Applied Pharmacology*.

[112] Koff JL, Shao MXG, Ueki IF, Nadel JA (2008). Multiple TLRs activate EGFR via a signaling cascade to produce innate immune responses in airway epithelium. *American Journal of Physiology*.

[113] Kuwahara I, Lillehoj EP, Lu W (2006). Neutrophil elastase induces IL-8 gene transcription and protein release through p38/NF-*κ*B activation via EGFR transactivation in a lung epithelial cell line. *American Journal of Physiology*.

[114] Pourazar J, Blomberg A, Kelly FJ (2008). Diesel exhaust increases EGFR and phosphorylated C-terminal Tyr 1173 in the bronchial epithelium. *Particle and Fibre Toxicology*.

[115] Burgel PR, Nadel JA (2008). Epidermal growth factor receptor-mediated innate immune responses and their roles in airway diseases. *European Respiratory Journal*.

[116] Jalava PI, Aakko-Saksa P, Murtonen T (2012). Toxicological properties of emission particles from heavy duty engines powered by conventional and bio-based diesel fuels and compressed natural gas. *Particle and Fibre Toxicology*.

[117] Nordenhäll C, Pourazar J, Blomberg A, Levin JO, Sandström T, Ädelroth E (2000). Airway inflammation following exposure to diesel exhaaust: a study of time kinetics using induced sputum. *European Respiratory Journal*.

[118] Salvi SS, Nordenhall C, Blomberg A (2000). Acute exposure to diesel exhaust increases IL-8 and GRO-*α* production in healthy human airways. *American Journal of Respiratory and Critical Care Medicine*.

[119] Li N, Hao M, Phalen RF, Hinds WC, Nel AE (2003). Particulate air pollutants and asthma: a paradigm for the role of oxidative stress in PM-induced adverse health effects. *Clinical Immunology*.

[120] Holder AL, Lucas D, Goth-goldstein R, Koshland CP (2008). Cellular response to diesel exhaust particles strongly depends on the exposure method. *Toxicological Sciences*.

[121] Li J, Ghio AJ, Cho SH, Brinckerhoff CE, Simon SA, Liedtke W (2009). Diesel exhaust particles activate the matrix-metalloproteinase-1 gene in human bronchial epithelial in a *β*-arrestin-dependent manner via activation of RAS. *Environmental Health Perspectives*.

[122] Cooney DJ, Hickey AJ (2011). Cellular response to the deposition of diesel exhaust particle aerosols onto human lung cells grown at the air-liquid interface by inertial impaction. *Toxicology in Vitro*.

